# Trochleoplasty Provides Good Outcomes for Recurrent Patellofemoral Dislocations with No Clear Superiority across Different Techniques

**DOI:** 10.3390/jcm13103009

**Published:** 2024-05-20

**Authors:** Sharon Si Heng Tan, Gin Way Law, Sunny Sunwoo Kim, Ervin Sethi, Andrew Kean Seng Lim, James Hoi Po Hui

**Affiliations:** Department of Orthopaedic Surgery, National University Health System, Singapore 119228, Singapore; sharon_sh_tan@nuhs.edu.sg (S.S.H.T.); sunnykim@u.nus.edu (S.S.K.); ervin.sethi@mohh.com.sg (E.S.); doslksa@nus.edu.sg (A.K.S.L.); doshuij@nus.edu.sg (J.H.P.H.)

**Keywords:** trochleoplasty, patella instability, trochlear dysplasia, patellofemoral instability

## Abstract

**Background:** Literature is sparse on outcome comparisons between different trochleoplasty techniques in the treatment of patella instability. To date, it is unclear whether there is a technique that offers superior outcomes. This systematic review and meta-analysis aims to compare and evaluate the outcomes of trochleoplasty techniques in the treatment of patellofemoral instability in trochlea dysplasia to establish whether there is an ideal choice of trochleoplasty technique for superior outcomes. **Methods**: 21 studies involving 880 knees were included. The mean age of the patients was 21.7 years (range 8–49 years). Mean follow-up timeframe of 43.5 months (range 8.8–100 months). Clinical outcomes assessed included rates of recurrence of patellofemoral dislocation, patient satisfaction, Kujala score, International Knee Documentation Committee (IKDC) score, Tegner score, and Lysholm score. Egger’s test showed no publication bias across all outcomes assessed. **Results**: Favourable results were seen across all outcomes assessed and patient satisfaction. Improvements were seen with Kujala, IKDC, and Lysholm scores. Tegner scores showed good return to function. Post-operative dislocation and complication rates were low across the different techniques. Meta-regression for Kujala and IKDC scores showed good outcomes regardless of trochleoplasty technique used (Kujala, *p* = 0.549, relative risk 492.06; IKDC, *p* = 0.193, RR 0.001). The exact risk that trochleoplasty poses to the cartilage remains uncertain, as no study had a conservatively managed arm for comparison. **Conclusions**: Trochleoplasty yielded good outcomes irrespective of technique used with no clear superiority demonstrated in any technique in terms of outcome scores, satisfaction, post-operative dislocation rates or complications.

## 1. Introduction

Trochleoplasty alters the kinematics of the patellofemoral joint by reshaping the bony architecture of the dysplastic trochlea to improve the congruency of the articulating surfaces in patients with recurrent patellofemoral dislocations. Despite improved outcomes and reduced dislocation rates with trochleoplasty [1,2,3,4,5,6], its use as a first-line treatment for patella instability remains controversial, primarily due to concerns about cartilage damage and the accelerated osteoarthritis associated with the procedure [2], and is commonly left as the last resort for surgical intervention when other options are exhausted.

The four main trochleoplasty techniques described in the literature are (1) a lateral facet elevating osteotomy with the interposition of a tibial graft to increase the obliquity of the trochlea, first described by Albee in 1915 [7]; (2) a sulcus deepening trochleoplasty proposed by Masse [8] and modified by Dejour [9], which uses a thick osteochondral flap to achieve the V-shaped groove [10]; (3) a thin malleable osteochondral flap [11] with the U-shaped trochleoplasty technique introduced by Bereiter [12]; and (4) a recession wedge trochleoplasty proposed by Goutallier to reduce the prominence of the trochlear bump, thereby decreasing the patellofemoral constraint and improving patella tracking to reduce lateral subluxation [13]. In recent years, these have been combined in various permutations with realignment or patella stabilising procedures, such as medial patellofemoral ligament (MPFL) reconstruction, medial reefing, medial tubercle transposition or vastus medialis obliquus (VMO) plasty [1,5,14,15,16,17,18,19].

To date, there are no randomised clinical trials involving trochleoplasty for the treatment of lateral patella instability. Given the multifactorial nature of patellofemoral instability, numerous surgical procedures are available, and the myriad of possible combinations with supplementary bony and soft tissue procedures, possible cartilage damage, and the potential of associated accelerated osteoarthritis. Analysis of the outcomes is complicated. Our systematic review and meta-analysis, therefore, aims to compare and evaluate the outcomes of trochleoplasty in patellofemoral instability to identify whether there is a superior choice of technique in trochleoplasty if reshaping of bony architecture for improved joint congruency is required in the management of patella instability.

## 2. Materials and Methods

### 2.1. Systematic Review

The systematic review was performed using the Preferred Reporting Items for Systematic Reviews and Meta-Analyses (PRISMA) guidelines. The search was conducted using PubMed, the Cumulative Index to Nursing and Allied Health Literature (CINAHL), and The Cochrane Library from inception through to 31 December 2017. The keywords used were ‘trochleoplasty’ or ‘trochleaplasty’ or ‘trochlear dysplasia’ or ‘patellofemoral instability’ or ‘patellar instability’ or ‘patella instability’.

All studies that reported the outcomes of trochleoplasty for recurrent patellofemoral dislocations were included. Studies where the patients did not have patellofemoral instability, studies where the patellofemoral instability was not managed with trochleoplasty, studies that did not report clinical outcomes, studies where the outcomes cannot be extracted for patients with trochleoplasty, studies with a sample size of less than ten, review articles, non-English articles, and articles with no full text available were excluded.

**Figure 1:**
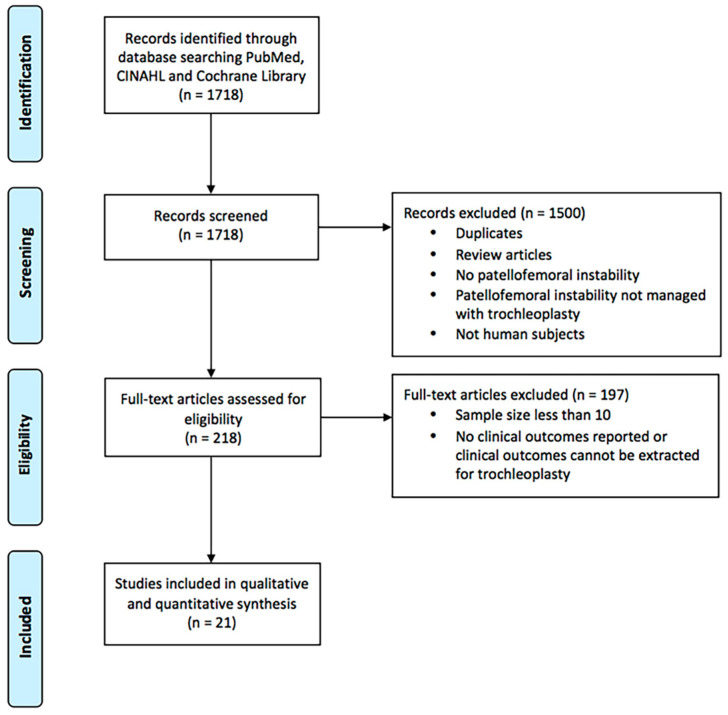
PRISMA flow diagram depicting the selection process for the systematic review and meta-analysis.

The articles were selected in two stages (Figure 1). First, the abstracts identified by the above searches were downloaded, and the list was screened using the inclusion and exclusion criteria. Second, the full texts of this shortlisted list were downloaded and assessed for eligibility. The reference lists of the publications were then hand-searched for additional relevant studies. This process was repeated twice independently. The articles identified were then assessed for level of evidence in accordance with the Oxford Centre of Evidence-Based Medicine.

### 2.2. Data Abstraction

Each study’s data was then retrieved individually. All clinical outcomes reported by three or more studies were included. These included the rates of recurrence of patellofemoral dislocation, patient satisfaction, Kujala score, International Knee Documentation Committee (IKDC) score, Tegner score, and Lysholm score. The surgical technique of the trochleoplasty was also noted.

The Kujala and IKDC scores are subjective patient-reported evaluation systems rated on a scale of 0 to 100 following knee injury. Specifically, the Kujala score assesses patellofemoral disorders in patients based on 6 activities regarded as triggers for anterior knee pain syndrome. The IKDC score assesses symptoms and function in daily living activities. Moreover, the Tegner and Lysholm scores are often jointly administered to evaluate sports and daily activity levels on a scale of 0 to 10, and subjective knee symptoms (e.g., pain and instability) out of 100, respectively [20]. Patient satisfaction was assessed via overall patient satisfaction in relation to the surgical procedures performed, via specific questionnaires, or extracted specifically from the patient-reported outcome scoring systems for assessment.

### 2.3. Data Analysis

The random effect model was used to analyse pooled estimates of pre-operative and post-operative differences for outcomes that were reported in three or more studies [21]. The random effect model assumes that the studies represented a random sample, with each study having its own underlying effect size. Under this model, it is assumed that there is a mean population-effect size about which the study-specific effect varies. As the random effects model properly takes into account the inter-study heterogeneity, such as differences in study design and definitions of outcomes, it provides a more conservative evaluation of the significance of the association than one based on fixed effects [22]. The pooled odds ratio (OR) or mean difference (MD) was then reported with a 95% confidence interval (CI). Forest plots were also provided.

Tests of heterogeneity were conducted while pooling the differences. This was done with the Q statistic that is distributed as a chi-square variate under the assumption of the homogeneity of effect sizes. The extent of between-study heterogeneity was assessed with the I2 statistic [23,24]. Meta-regression was performed when the overall outcomes were heterogeneous. This identifies the moderators that might contribute to the heterogeneity of the effect sizes. Study identifiers were added to the model to control for the effect of any variations in study characteristics. The regression coefficient was calculated to indicate the percentage of variance explained by the moderators, and significant moderators were reported together with the associated adjusted pooled relative risk estimate with a 95% CI.

Egger’s statistical tests were also conducted to evaluate the possibility of publication bias for the outcomes analysed [25].

All statistical evaluations were made assuming a two-sided test at the 5% level of significance using Stata version 12 (Stata Corp, College Station, TX, USA).

## 3. Results

Twenty-one studies involving 881 knees met our criteria for assessment [4,11,17,18,19,26,27,28,29,30,31,32,33,34,35,36,37,38,39,40,41]. The mean age of the patients was 21.7 years (range 8–49 years) with a mean follow-up timeframe of 43.5 months (range 8.8–100 months). Patient demographics, type of trochleoplasty, and follow-up duration for each study are detailed in Table 1.

All 21 studies in our systematic review were performed in Europe, with the thin flap U-shaped trochleoplasty technique most commonly performed (57.1%), followed by the sulcus deepening trochleoplasty with the thick osteochondral flap (28.6%), the lateral facet elevating trochleoplasty (9.5%), and recession wedge trochleoplasty (4.8%), respectively.

The exclusion criteria for the individual studies included pregnant patients, patients with open epiphyseal plates, patients under 15 years of age, the presence of patellofemoral or rheumatic arthritis, other systemic diseases, patellofemoral pain syndrome with no true dislocation, previous lower-limb operations or knee fractures, additional surgical interventions, e.g., osteotomies and tibial tuberosity transfers), degenerative changes of trochlear cartilage, or habitual patellar dislocations due to femoral malrotation. Patients with incomplete clinical and/or radiographic medical charts, as well as those unavailable for follow-up in an outpatient clinic, were also excluded. The specific inclusion and exclusion criteria of each study can be found in the Supplementary Materials.

### 3.1. Quality of Studies

Six of the 21 studies were prospective studies; the remaining 15 studies were retrospective studies. All studies had Level 4 evidence. Egger’s test showed no publication bias across all the outcomes assessed (Table 2).

### 3.2. Outcome Scores

**Figure 2:**
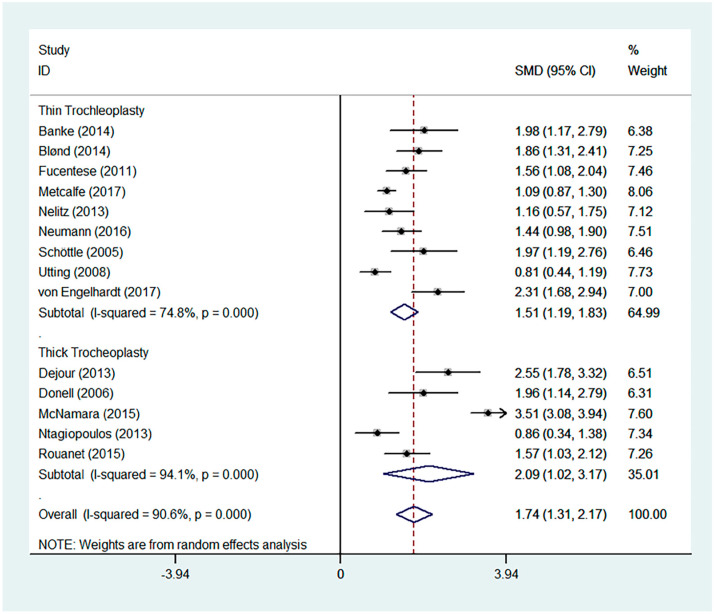
Forest plot for Kujala score [4,17,18,19,26,27,29,30,32,34,35,37,38,40].

**Figure 3:**
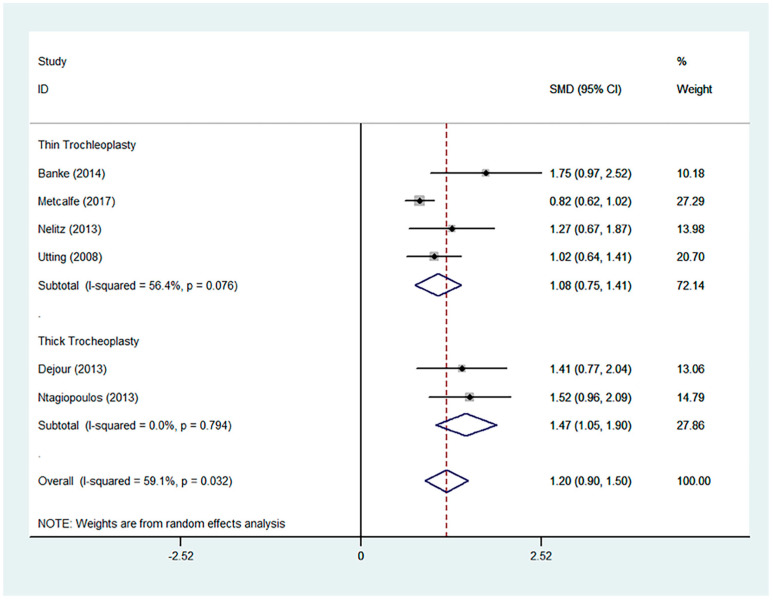
Forest plot for IKDC score [4,18,26,29,34,37,40].

**Figure 4:**
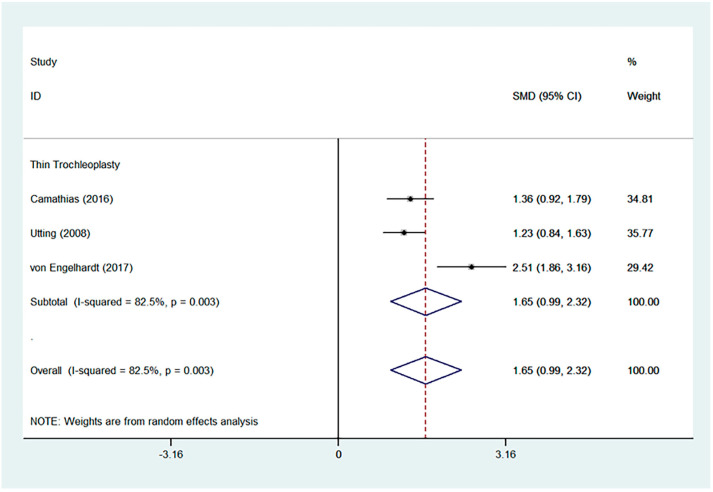
Forest plot for Lysholm score [19,28,40].

**Figure 5:**
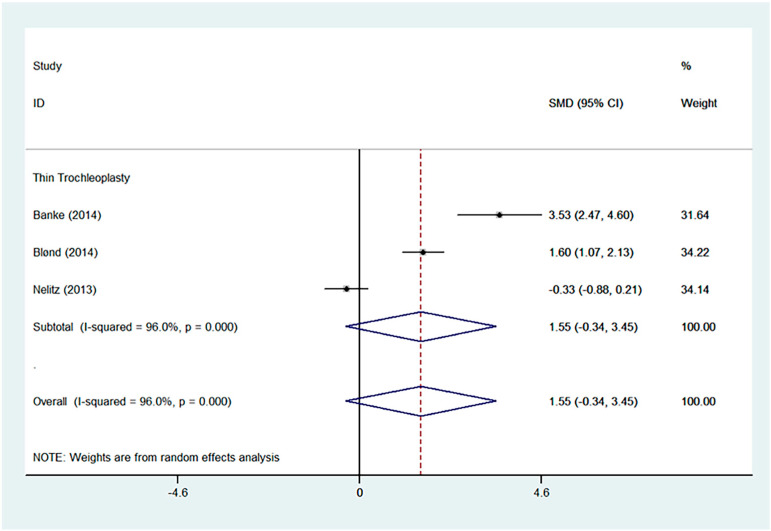
Forest plot for Tegner score [18,26,27].

**Figure 6:**
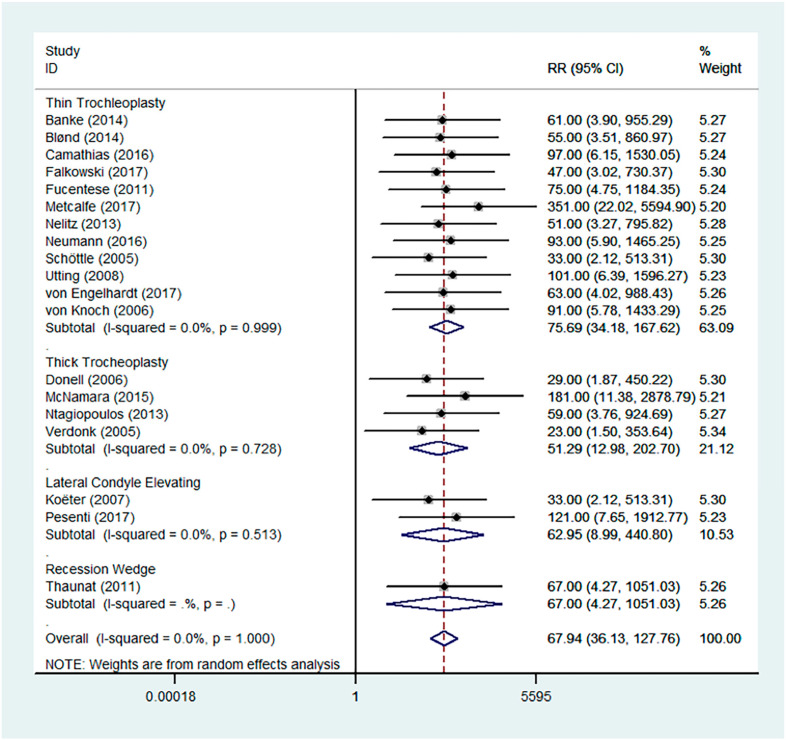
Forest plot for patient satisfaction [4,11,17,18,19,26,27,28,30,31,32,33,34,35,36,38,39,40,41].

Favourable results were seen across all outcomes scores assessed with significant improvements in Kujala, IKDC, and Lysholm scores (Figure 2, Figure 3 and Figure 4). Tegner scores showed a good return to function, with no significant difference in post-operative scores compared to pre-operative scores (Figure 5). Patient satisfaction was excellent and consistent across the different trochleoplasty techniques (Figure 6).

The overall results were heterogeneous for the Kujala, IKDC, Lysholm and Tegner scores (Table 2). Meta-regressions for the Kujala and IKDC scores showed good outcomes regardless of the trochleoplasty technique used (Kujala, *p* = 0.549, relative risk 492.06; IKDC, *p* = 0.193, RR 0.001). Meta-regressions were not performed for Lysholm and Tegner scores even though the results were heterogeneous because the same surgical technique was used.

### 3.3. Complications

**Figure 7:**
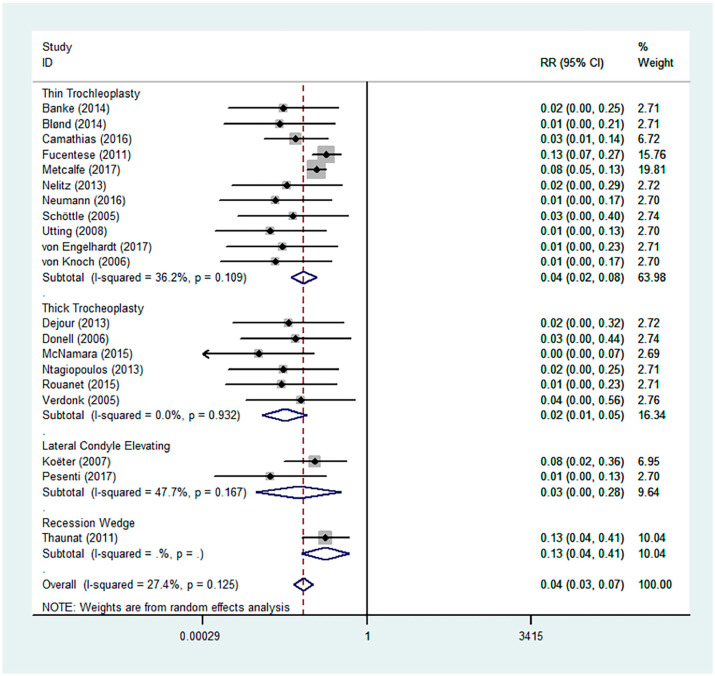
Forest plot for patellofemoral dislocation recurrence rates [4,11,17,18,19,26,27,28,29,30,32,33,34,35,36,37,38,39,40,41].

Post-operative patellofemoral dislocation recurrence rates were low and similar across the different techniques (Figure 7). Complication profiles, including post-operative pain, residual symptoms and signs, and the re-operation rates of the different studies, are shown in Table 3.

Patellofemoral dislocation recurrences were reported in 20 of the 21 studies. This occurred in 18 of the 568 knees (3.2%) that underwent thin flap U-shaped trochleoplasty, 0 of the 226 knees (0%) that underwent sulcus deepening trochleoplasty with the thick osteochondral flap, 0 of the 46 knees (0%) that underwent lateral facet elevating trochleoplasty, and 2 out of the 19 knees (10.5%) that underwent recession wedge trochleoplasty.

Development or progression of preexisting patellofemoral osteoarthritis was reported in 64 knees, of which 25 underwent thin flap U-shaped trochleoplasty, 33 underwent sulcus deepening trochleoplasty with the thick osteochondral flap, and 6 underwent lateral facet elevating trochleoplasty. Eight of the 64 knees required revision to arthroplasty (4 patellofemoral arthoplasty and 4 total knee replacements).

Rates of infection were low, with six cases of superficial wound infections and no cases of deep infections reported.

Re-operation rates were significant, ranging from 1.7–17.2% for thin flap U-shaped trochleoplasty, 4.2–76.5% sulcus deepening trochleoplasty with the thick osteochondral flap, 3.3% in lateral facet elevating trochleoplasty, and 63.2% in recession wedge trochleoplasty, respectively.

The reasons for re-operations include arthrofibrosis, overtightening, removal of loose bodies or implants, symptomatic subluxation or dislocation, and osteoarthritis. Revision to arthroplasty was performed in eight knees (four patellofemoral arthoplasty and four total knee replacements).

Less common complications reported include transient postoperative femoral nerve palsy after peripheral anaesthesia (one patient), CRPS (one patient), deep vein thrombosis (two patients), pulmonary embolism (one patient), anaphylaxis to prophylactic antibiotic (one patient), patella baja (one patient), poor wound healing (one patient), and postoperative haematoma (one patient).

Fifty-one patients reported some residual instability in the knee. The J-sign was positive in eight patients.

## 4. Discussion

The key finding from our systematic review of 21 studies involving 881 knees on the different trochleoplasty techniques was that trochleoplasty yielded good outcomes irrespective of the technique used, with consistent results demonstrated across all techniques in terms of outcome scores, patient satisfaction, and post-operative dislocation rates. Longo et al.’s systematic review was the only study that compared the outcomes of different trochleoplasty procedures. They included 392 knees and found the lowest rates of post-operative patellar redislocation, osteoarthritis, and deficiency in range of motion with the Bereiter U-shaped deepening trochleoplasty and the highest mean post-operative Kujala scores with the Dejour V-shaped deepening trochleoplasty [3].

Our systematic review assessed a significantly larger number of studies and knees compared to Longo et al.’s study and was more comprehensive, as we considered all the different outcomes reported across the studies. We also evaluated the impact of inter-study heterogeneity, as well as the potential moderators for study heterogeneity so as to better control their effect on variations in the study characteristics to provide a more robust comparison and accurate review of the different trochleoplasty techniques. Nonetheless, the difficulty in making definitive conclusions on outcomes of trochleoplasty lies in the heterogeneity of the data in the literature due to inconsistent reporting of outcome measures, complication profiles, and residual symptoms, on top of the multitude of permutations with different trochleoplasty techniques and supplementary procedures.

Patients with high-grade trochlear dysplasia have a high rate of progression to patellofemoral osteoarthritis over time [42,43]. As cartilage functions as a signalling scaffold, early iatrogenic to cartilage may lead to the subsequent development of osteoarthritis through the release of bioactive matrix components and soluble factors that can interact with chondrocytes to cause inflammation, loss of phenotypic stability, and further degradation of the cartilage extracellular matrix [44]. These can contribute to the aggressive and deleterious course of osteoarthritic disease through the continued release of these cartilage degradation mediators with increased cartilage damage, which ultimately results in progressive remodelling and osteoarthritic change. Compared to patients with stable patellae, patients with lateral patella instability also have a higher incidence of cartilage lesions and degenerative wear [28,45,46,47], likely due to prolonged overloading over time. Much of the controversy in trochleoplasty stems from concerns about cartilage damage and accelerated patellofemoral arthritis associated with the procedure [2]. However, given the known associations between trochlear dysplasia and osteoarthritis in the native knee, demonstrating any additional risk to cartilage conferred by trochleoplasty will require matched-pair studies with similar degrees of dysplasia, similar pre-existing chondral damage, and similar patient profiles in both the trochleoplasty and non-operative arms, with similar activity levels subsequently over the follow-up timeframe to control wear rates, given the degenerative aetiology of osteoarthritis.

Progression of osteoarthritis was reported in 5 of the 21 studies in our review [11,33,35,36,37]. In particular, von Knoch et al. reported progression of patellofemoral osteoarthritis to Iwano grade 2 or more in 10 of the 33 patients that underwent the same thin flap trochleoplasty procedure over the follow-up timeframe of 8.3 years (range 4–14 years) [11]. Additionally, Rouanet et al. reported progression of patellofemoral osteoarthritis to Iwano grade 3 or more in 20 of the 34 knees that underwent the sulcus deepening trochleoplasty with the thick osteochondral flap over their mean follow-up timeframe of 15.3 years (12–19 years) [37]. Although the progression of patellofemoral arthritis was demonstrated in these studies, none had a conservatively managed comparative arm to differentiate the impact of trochleoplasty on cartilage wear from the natural progression of patellofemoral osteoarthritis in patients with dysplastic trochleae and persistent instability with recurrent dislocation. While trochleoplasty may have an impact on patellofemoral wear, the evidence to date does not definitively show whether there is increased risk to the cartilage with the procedure compared to conservative management and will not be clinically relevant if the patient is asymptomatic.

Patient-reported outcome measures (PROM) have become a cornerstone in the assessment of outcomes post-surgery [48,49,50,51,52,53,54,55,56]. Although Kujala, IKDC, Lysholm, and Tegner scores were not designed specifically for the assessment of lateral patella instability, they were widely accepted scoring systems used at the times the surgeries were performed and are useful as a comparative outcome measure rather than an absolute measurement of outcome. The combination of excellent patient satisfaction across all trochleoplasty techniques and favourable results consistent across all outcome scores assessed (Kujala, IKDC, Lysholm, and Tegner scores) sends a clear message from patients that trochleoplasty improved their quality of life. Nonetheless, future studies with disease-specific PROM, such as the Banff Patella Instability Instrument (BPII) and Norwich Patellar Instability Score (NPI), validated recently [57] will be useful in the evaluation of treatment outcomes in patients with lateral patella instability to quantify the degree of improvement and to further delineate intricate differences between the trochleoplasty techniques.

Though patellofemoral dislocation recurrence rates were low [0.04 (95% CI 0.03–0.07)] over the mean follow-up timeframe of 43.5 months, re-operation rates were significant. This was especially apparent in the recession wedge trochleoplasty, where 12 out of 19 knees (63.2%) had revision surgeries, as well as in sulcus deepening trochleoplasty with thick osteochondral flap (4.2–76.5%). Accordingly, this may suggest underlying flaws associated with these techniques, including residual instability and corresponding complications. Nonetheless, given that only one paper using recession wedge trochleoplasty was included in our review, inherent potential biases must be taken into consideration.

The outcomes of trochleoplasty have also been compared with other patella stabilising procedures. In Hiemstra et al.’s systematic review of trochleoplasty performed in 998 patients for lateral patellofemoral instability, they concluded that trochleoplasty results in good clinical outcomes, a low re-dislocation rate, and an acceptable complication profile in both short and long-term follow-up in patients with high-grade trochlear dysplasia [2]. In Balcarek et al.’s systemic review involving 407 knees, they also found that trochleoplasty with extensor balancing yielded superior results in the prevention of subsequent post-operative dislocation/subluxation compared to MPFL reconstruction alone in severe trochlear dysplasia [1].

This is one of the few systematic reviews and meta-analyses examining the surgical trends and outcomes of trochleoplasty. This study adds data in an area not well understood and aids in clinical counselling for patients considering trochleoplasty for patellofemoral instability. Our study limitations include (1) inherent selection bias from the retrospective study design in the studies included, (2) heterogeneity of the reported studies, and (3) limited generalizability to all populations globally, given that all studies were performed in Europe.

Confounded by the multifactorial aetiology of patellofemoral instability, lack of adequately powered studies and numerous possible permutations with supplementary procedures, the current knowledge on the ideal choice of trochleoplasty technique to address patellofemoral instability is still in its infancy. Further studies with a more comprehensive pre-operative assessment of pre-existing chondral lesions or degenerative wear, type of trochlear dysplasia, and standardized reporting criteria for outcomes will help identify whether there is an ideal timing for intervention and whether there is an ideal trochleoplasty technique or ideal combination with supplementary procedures in the management of patella instability.

## 5. Conclusions

The heterogeneity of the data in the literature in terms of reporting of outcome measures, post-procedure residual symptoms, definitions of complications, and a multitude of possible permutations with different supplementary procedures used has made comparisons between different trochleoplasty techniques challenging.

The thin flap U-shaped trochleoplasty remains the most commonly performed and well-studied trochleoplasty technique. This systematic review and meta-analysis has identified that, while there are concerns about the risk of iatrogenic cartilage damage and possible accelerated osteoarthritis associated with trochleoplasty, trochleoplasty remains an appropriate surgical intervention for patellofemoral instability with trochlea dysplasia. Irrespective of the technique used with low patellofemoral dislocation recurrence rates, good clinical outcomes are evidenced by well-established patient-reported outcome scores and an acceptable complication profile.

## Figures and Tables

**Table 1:** Details of the studies included in the review.

Author	Year	Level of Evidence	Sample Size (Number of Knees)	Type of Trocheoplasty	Age (Years)			Sex *		Follow Up (Months)		
					Mean	Minimum	Maximum	Male	Female	Mean	Minimum	Maximum
Banke	2014	IV	18	Thin Trochleoplasty	22.2	15	31	6	11	30.5	24	40
Blønd	2014	IV	29	Thin Trochleoplasty	19	12	39	10	21	29	12	57
Camathias	2016	IV	50	Thin Trochleoplasty	15.6	13	20.4	20	30	33	24	64
Dejour	2013	IV	24	Thick Trocheoplasty	23	14	33	9	13	66	24	191
Donell	2006	IV	17	Thick Trocheoplasty	25	15	47	3	12	36	12	108
Falkowski	2017	IV	22	Thin Trochleoplasty	16.3	13.9	19	4	18	8.8	3	12
Fucentese	2011	IV	44	Thin Trochleoplasty	18	14	40	10	28	48	24	93.6
Koëter	2007	IV	19	Lateral Condyle Elevating	25	15	34	4	12	51	24	110
McNamara	2015	IV	107	Thick Trocheoplasty	23	12	49	36	54	72	24	228
Metcalfe	2017	IV	199	Thin Trochleoplasty	21.3	14	38	52	133	53.16	12	144
Nelitz	2013	IV	26	Thin Trochleoplasty	19.2	15.4	23.6	14	9	36	24	42
Neumann	2016	IV	46	Thin Trochleoplasty	27.6 ^#^	16	53	13	33	56.7 ^#^	24	109
Ntagiopoulos	2013	IV	31	Thick Trocheoplasty	21	14	47	14	13	84	24	108
Pesenti	2017	IV	27	Lateral Condyle Elevating	12.5	8	17	11	12	NR	60	NR
Rouanet	2015	IV	34	Thick Trocheoplasty	27.8	16	49	10	24	183.6	144	228
Schöttle	2005	IV	19	Thin Trochleoplasty	22	17	40	3	13	36	24	48
Thaunat	2011	IV	19	Recession Wedge	23	18	45	8	9	34	12	71
Utting	2008	IV	59	Thin Trochleoplasty	21.5	14.3	33.9	15	39	24	12	58
Verdonk	2005	IV	13	Thick Trocheoplasty	27	14	39	3	9	18	8	34
von Engelhardt	2017	IV	33	Thin Trochleoplasty	24	SD 9 ^+^	SD 9 ^+^	12	21	29	SD 23 ^+^	SD 23 ^+^
von Knoch	2006	IV	45	Thin Trochleoplasty	22.2	15	31	16	22	]	48	168

NR: Not Reported; * Number of males and females are reported as per number of patients and not as per number of knees; ^+^ Distribution is reported as standard deviation (SD) instead of minimum and maximum; ^#^ Value is reported as median instead of mean.

**Table 2:** Meta-analysis, tests for heterogeneity and Egger’s test for publication bias.

Outcomes	Meta-Analysis					Tests for Heterogeneity		Egger’s Test
	Pooled Estimate		95% Confidence Interval			*p*-Value	I^2^	*p*-Value
Kujala	SMD	1.74	1.31	-	2.17	0.000	90.6%	0.158
IKDC	SMD	1.20	0.90	-	1.50	0.032	59.1%	0.169
Tegner	SMD	1.55	−0.34	-	3.45	0.000	96.0%	0.828
Lysholm	SMD	1.65	0.99	-	2.32	0.003	82.5%	0.960
Dislocation	RR	0.04	0.03	-	0.07	0.125	27.4%	0.999
Satisfaction	RR	67.94	36.13	-	127.76	1.000	0.0%	0.999

**Table 3:** Post-operative pain, residual symptoms and signs, complication profile, and re-operation rate.

Author	Year	Sample Size (Number of Knees)	Pain	Residual Symptoms and Signs	Complications	Re-Operations
Banke	2014	18	VAS 5.6 (2.8) to 2.5 (1.7)	NR	1 over tight MPFLR (5.6%) 2 arthrofibrosis (11.1%)	1 re-tension MPFLR 2 arthroscopic arthrolysis
Blønd	2014	29	NR	2 residual instability, J sign positive (6.9%)	2 symptomatic subluxations (6.9%) 3 anterior knee pain secondary to tight lateral retinaculum (10.3%)	2 medialisation of tibial tubercle 3 lateral release
Camathias	2016	50	NR	6 J sign positive (12%) 8 apprehension positive (16%)	1 dislocation (2%) 4 arthrofibrosis (8%)	1 revision with retrochleoplasty, MPFL-plasty 4 arthroscopic arthrolysis
Dejour	2013	24	Pain decreased in 72% of cases, unchanged, or increased in 28%	6 apprehension positive (25%)	No patellofemoral osteoarthritis No postoperative stiffness No dislocation	1 removal of hardware after staple breakage
Donell	2006	17	NR	7 apprehension positive	11 crepitus	5 arthroscopic arthrolysis 1 re-medial reefing 1 patellar chondroplasty 1 autologous chondrocyte implantation in lateral femoral condyle 1 removal of loose screw head 4 removal of screws only
Falkowski	2017	22	NR	6 apprehension positive	NR	NR
Fucentese	2011	44	VAS 8 (3–10) to 8 (3–10); *p* = 0.027)	11 apprehension positive (25%) 11 residual instability (25%)	1 dislocation (2.3%) 1 transient postoperative femoral nerve palsy after peripheral anaesthesia (2.3%) 1 poor wound healing (2.3%) 1 CRPS (2.3%)	1 MPFL reconstruction 1 anteromedialization of tibial tuberosity
Koëter	2007	19	13 patients reported pain relieved at rest 12 patients reported pain relieved during activities	1 residual instability	2 progression of osteoarthritis 1 post-operative haematoma 2 subluxation after rotational trauma 1 failure (persisting pain requiring revision arthroplasty) No arthrofibrosis	1 patellofemoral arthroplasty 1 evacuation of post-operative haematoma 1 tibial tubercle repositioning
McNamara	2015	107	34%	34.3–74.5% apprehension positive 12 residual instability	1 DVT 1 pulmonary embolism 8 arthrofibrosis 4 superficial wound infection 4 crepitus	10 MPFL-R 8 arthrolysis 2 removal of loose screw head 1 arthroscopic debridement 2 patelloplasty
Metcalfe	2017	199	25% had residual pain	12 residual instability 2 quadriceps weakness	16 dislocation 2 arthrofibrosis 1 over tight MPFLR 1 partial detachment of cartilage flap 1 recurrent knee effusion 2 intraarticular loose bodies 1 CRPS 1 foot drop	9 MPFLR 7 TTO 2 MUA 1 release of tight MPFL reconstruction 6 arthroscopy 2 removal of TTO screw
Nelitz	2013	26	VAS 3 (1–7) to 1 (0–5); *p* =< 0.01	1 apprehension positive	1 poor post-operative knee flexion requiring prolonged rehabilitation to achieve full range of motion No recurrent dislocation No wound infection 3 patellofemoral crepitus	NR
Neumann	2014	46	NR	No apprehension	No dislocation 3 with preexisting patellofemoral osteoarthritis showed radiological progression of osteoarthritis	NR
Ntagiopoulos	2013	31	75% reported decrease in pain	No residual instability 6 apprehension positive	No dislocation recurrence 2 hardware (staple) breakage 1 DVT No patellofemoral arthritis	2 arthroscopic removal of hardware
Pesenti	2017	27	3 had occasional knee pain after prolonged physical activity. The rest of the patients were pain free on follow up.	2 residual instability	No dislocation 4 developed lateral patellofemoral osteoarthritis	NR
Rouanet	2015	34	Out of 27 patients without revision, 18 had no pain or only occasional pain, 1 had significant pain	Out of 27 patients without revision, 10 residual instability 3 apprehension positive	7 failures (6 osteoarthritis, 1 gives way frequently) 8 arthrofibrosis Pre-operatively, 10 had patellofemoral osteoarthritis (none > Iwano 2) Post-operatively, 33 had patellofemoral osteoarthritis [20 (65%) > Iwano 2)]	3 total knee arthroplasty 3 patellofemoral arthroplasty 1 tibial tubercle transfer 6 MUA 2 arthroscopic release
Schöttle	2005	19	Pain improved in 12 knees and worsened in 2 knees	4 apprehension positive	No dislocation	NR
Thaunat	2011	19	All but 1 patient had slight pain on follow up. Pain was generally localised at the level of the tibial tubercle screw site for those operated for pain-free instability. Significant pain improvement was reported in all but one patient for those with pain preoperatively.	6 apprehension positive (31.6%)	2 dislocation (10.5%) 1 arthrofibrosis (5.3%) 9 patellofemoral crepitus (50.0%)	1 arthroscopic arthrolysis 1 arthroscopic supratrochlear exostosectomy 8 removal of screws from anterior tibial tubercle and trochlea 2 for tibial tubercle pseudoarthrosis
Utting	2008	59	8 had residual pain	8 had continued swelling or crepitation (14.8%) No recurrent instability	2 superficial infection 1 arthrofibrosis 1 traumatic dislocation 1 anaphylaxis to prophylactic antibiotic	1 MUA
Verdonk	2005	13	2 persistent retropatellar pain (15.4%)	7 patellofemoral crepitus (53.8%)	No dislocation 5 arthrofibrosis 3 impingement of fixation material 1 complete failure	5 MUA 3 arthroscopic removal of fixation material 1 total knee arthroplasty
von Engelhardt	2017	33	VAS 4.8 (2.0) to 1.3 (3.4); *p* < 0.0001)	2 avoidance behaviour (6.1%)	5 arthrofibrosis (15.2%)	2 arthroscopic arthrolysis (6.1%)
von Knoch	2006	45	Post-operative pain increased in 15 knees (33.4%), remained unchanged in 4 (8.8%) and improved in 22 (49%). 4 knees (8.8%) which were pain free pre-operatively remained pain free post-operatively	No apprehension 1 residual instability	1 patella baja No dislocation Development of patellofemoral osteoarthritis in 22 of 31 knees (72.7%) and tibiofemoral osteoarthritis in 4 of 33 knees (15.2%) with no pre-existing osteoarthritis radiologically prior to surgery 28 patellofemoral crepitus (62.2%)	1 Elmslie–Trillat procedure for distal realignment

MPFLR: medial patellofemoral ligament reconstruction; CRPS: chronic regional pain syndrome; RPD: recurrent patella dislocation; TD: trochlear dysplasia; MUA: manipulation under anaesthesia; NR: not reported; DVT: deep vein thrombosis.

## Data Availability

The original contributions presented in the study are included in the article; further inquiries can be directed to the corresponding author.

## Supplementary Materials

The following supporting information can be downloaded at: https://www.mdpi.com/article/10.3390/jcm13103009/s1, Table S1: Inclusion and exclusion criteria of the studies included in the review.
