# Supplementary material for: Trochleoplasty Provides Good Outcomes for Recurrent Patellofemoral Dislocations with No Clear Superiority across Different Techniques
Source: J Clin Med. 2024 May 20;13(10):3009. doi: 10.3390/jcm13103009 (PMC11122057; doi:10.3390/jcm13103009)
Supplement: Supplementary file 1 [file jcm-13-03009-s001.zip › jcm-2916289-supplementary.pdf]

Supplementary material

Supplementary Table S1: Inclusion and exclusion criteria of the studies included in the

Author	Year	Inclusion Criteria	Exclusion Criteria
Banke	2014	History of two or more patellar redislocations with a persistent positive apprehension sign from extension to 60 of flexion and a concomitant approval of trochlear dysplasia type B, C or D in the axial MRI undergoing trochleoplasty in combination with MPFLR by one surgeon (senior author)	Age below 15 years<br>Open epiphyseal plates<br>Pregnancy<br>Previous ligamentous surgeries at the affected knee<br>Patellofemoral arthritis<br>rheumatic arthritis<br>Habitual patella dislocation during the complete range of motion due to femoral malrotation
Blønd	2014	Patients with RPD and TD type B to D<br>Positive patella apprehension sign at a minimum of 20 flexion	None
Camathias	2016	Adolescent patient aged 10 to 20 years<br>Recurrent patellar dislocations not responding to at least 6 months of nonoperative treatment, minimum type B dysplasia<br>closed or closing physes (grade V or VI according with grade V indicating <5 mm on any single section on MRI and grade VI indicating completely fused physis).	Previous lower limb operations<br>Systemic diseases<br>Open physis (less than grade V)<br>Patient with additional surgical interventions (osteotomies for torsional or valgus/varus correction)
Dejour	2013	Skeletally mature patients with recurrent patellar dislocation<br>Presence of trochlear dysplasia<br>History of previous surgery for the treatment of patellar dislocation	Patients with patellofemoral arthritis<br>Patients with patellofemoral pain syndrome with no documented patellar dislocation
Donell	2006	Patients with recurrent dislocation of the patella with severe trochlear dysplasia	None
Falkowski	2017	Patients with clinical diagnosis of patellar instability, as determined by review of clinical records, who received MRI before and after surgery for treatment of patellar instability	None
Fucentese	2011	Trochleoplasty performed for patients with two or more patellar dislocations, or one patellar dislocation with a persistently positive patellar apprehension sign for more than 1 year in the presence of radiologically confirmed trochlear dysplasia	Radiological signs of arthritic degeneration of the patellofemoral joint<br>Open epiphyseal plates

Koëter	2007	Patients with objective patellar instability due to isolated trochlear dysplasia (as established on a true lateral radiograph) and closed epiphysis	Patients with instability due to patella alta, high Q angle, genu valgum, VMO dysplasia were treated with different operations
McNamara	2015	Patients with recurrent symptomatic patellar instability, with at least 1 documented dislocation of the patella that had either failed physical therapy and/or a previous patellar stabilization procedure(s), with severe trochlear dysplasia as defined on a lateral plain radiograph with a boss height of 4 mm	Patellofemoral arthritis<br>Patients who underwent a different type of trochleoplasty procedure (Bereiter, n = 2; modified Albee, n = 1; excision, n = 3)
Metcalfe	2017	Recurrent patellar instability (the sole clinical indication) and severe trochlear dysplasia (the sole radiological indication)	None
Nelitz	2013	Patients with severe trochlear dysplasia who had experienced at least 2 recurrent dislocations of the patella despite a nonoperative treatment program	Patients with a tibial tubercle–trochlear groove (TT-TG) distance >2.2 cm<br>International Cartilage Repair Society (ICRS) grade III or IV degenerative changes of the femoral trochlear cartilage<br>Patients who had additional varization osteotomy performed
Neumann	2016	Patients with repetitive but a minimum of two patella luxations<br>Previous non-operative treatment without clinical or subjective improvement, and reports of continual discomfort, pain, and instability of the affected knee joint<br>Patients with symptomatic but mild trochlear dysplasia	Incomplete clinical and/or radiographic medical charts<br>Unavailability for follow-up in outpatient clinic
Ntagiopoulos	2013	Patients with more than 3 documented episodes of patellar dislocation with high-grade trochlear dysplasia type B or D according to the Dejour classification<br>No previous patellofemoral surgery<br>Pathological lateral patellar glide test using the quadrant test (the patella can be shifted by 3 or more quadrants laterally by the examiner with the patient's knee placed at full extension)	Patients with open growth plates, patellofemoral arthritis, or patellofemoral pain syndrome with no true dislocation
Pesenti	2017	Presence of habitual or permanent patellar dislocations, which were radiographically documented, related to a grade B trochlear dysplasia	None

Rouanet	2015	Patients who presented with objective (patellofemoral dislocations) or subjective (apprehension without dislocation) patellofemoral instability associated with trochlear dysplasia	Adolescents with open growth plates<br>Painful patellar syndromes without clinical instability<br>Iwanoet al.'s stage ≥ 2 patellofemoral osteoarthritis
Schöttle	2005	2 or more patellar dislocations<br>1 patellar dislocation with a persistent<br>Fairbank's apprehension sign of more than 1 year in the presence of all radiological signs of trochlear dysplasia	Degenerative deformation of the trochlea Open epiphyseal plates
Thaunat	2011	Patellofemoral instability defined by recurrent subluxations or recurrent dislocations associated with dysplasia of the femoral trochlea.<br>Persistent retropatellar pain and crepitus due to trochlear dysplasia<br>Apprehension without dislocation<br>Failure to respond to conservative treatment	None
Utting	2008	Patellofemoral instability or chronic dislocation associated with dysplasia of the femoral trochlea	None
Verdonk	2005	Persistent retropatellar pain and crepitus due to trochleodyplasia, whether or not in combination with patella alta<br>Recurrent patellar dislocations	None
von Engelhardt	2017	Patients who underwent surgery with a combination of trochleoplasty and MPFL reconstruction with clear positive patella<br>apprehension sign prior to surgery	Additional surgeries such as corrections of the knee rotation and/or axis, tibial tuberosity transfers, etc.<br>Patients with advanced osteoarthritic changes<br>Previous fractures of the knee
von Knoch	2006	Recurrent patellar dislocation in the presence of a positive Fairbank's apprehension sign<br>Confirmation of trochlear dysplasia on radiographs	Open epiphyseal growth plates<br>Pronounced degenerative changes of the trochlea despite patellofemoral instability in association with trochlear dysplasia

MPFLR: medial patellofemoral ligament reconstruction

MRI: Magnetic Resonance Imaging

RPD: Recurrent patella dislocation

TD: trochlear dysplasia
